# Case series of family‐based treatment for restrictive‐type eating disorders and comorbid autism: What can we learn? A brief report

**DOI:** 10.1002/erv.2938

**Published:** 2022-07-08

**Authors:** Mette Bentz, Signe Holm Pedersen, Ulla Moslet

**Affiliations:** ^1^ Child and Adolescent Mental Health Center Copenhagen University Hospital – Mental Health Services CPH Copenhagen Denmark

**Keywords:** autism, restrictive‐type eating disorder, youth

## Abstract

**Objective:**

Autism is more prevalent among persons with Restrictive type eating disorders (R‐ED) compared to the general population and is associated with poorer outcomes across treatment modalities. Knowledge is sparse with regard to whether poorer outcomes are also associated with Family‐based treatment (FBT), which is recommended as the first choice of treatment for young persons (YPs) with R‐ED.

This case series compares outcome between groups with and without autism in a large consecutive series of YPs with R‐ED treated with FBT.

**Method:**

In an earlier described consecutive series of 157 YPs with R‐ED treated with FBT, we compared the outcomes of the subgroup with (*N* = 16) and without (*N* = 141) comorbid autism. Primary ICD‐10 diagnoses were typical (50.0) or atypical anorexia nervosa (AN) (F50.1), the latter implying a condition as typical AN but with a failure to meet one of the diagnostic criteria. Autism diagnoses were clinically assigned. The outcomes were receiving intensified care, weight normalisation and overall successful treatment.

**Results:**

10.2% (*N* = 16) of the sample had autism. 2.5% (*N* = 4) had autism diagnosed prior to the Eating Disorder (ED), and an additional 7.7% (*N* = 12) were diagnosed with autism during ED treatment. Significantly more YPs with autism (50%, *N* = 8) compared with YPs without autism (16%, *N* = 23) received intensified care (day programme or inpatient treatment) during their treatment. No significant difference between groups regarding neither weight normalisation nor successful ending of the treatment were found.

**Conclusion:**

This small sample of YPs with autism suggests that comparable proportions of YPs with and without autism may restore normal weight and end the treatment successfully within 12 months. However, more YPs with comorbid autism needed more intensive treatment, indicating that outpatient treatment delivery may not be sufficient to bring about desired change in this patient group. Findings need confirmation in a larger sample with a systematic screening for autism.

## Highlights

- 10% of young people with restrictive‐type eating disorder (R‐ED) had comorbid autism.
- More young people with R‐ED and comorbid autism need intensified treatment.
- Comparable proportions of young people with R‐ED with and without autism achieved weight normalisation and ended the treatment within 12 months.
- FBT may be a suitable first line of treatment for young persons (YPs) with R‐ED and autism, but adaptations might help more YPs recover with an outpatient modality alone.

## 1 INTRODUCTION AND AIMS

Restrictive‐type eating disorders (R‐ED) such as anorexia nervosa (AN) is a serious psychiatric disorder most often beginning in adolescence (Steinhausen & Jensen, 2015). Autism spectrum conditions are neurodevelopmental conditions defined by difficulties in social interaction and a tendency towards restricted, inflexible, or stereotypic interests or behaviour, manifested early in development (WHO, 2021). In this short report, we will use the term autism for the broad spectrum of autism spectrum conditions, in line with recommendations from Autism Europe (Autism Europe, 2022). Compared to the general population, autism is more prevalent among persons with R‐ED, as reviews find 4%–10% among YPs with R‐ED, and even higher prevalence in adult samples (Huke et al., 2013; Westwood & Tchanturia, 2017). The elevated prevalence suggests that autism may represent a vulnerability factor for R‐ED, and this is confirmed by individuals with a lived experience of both conditions (Brede et al., 2020). The presence of autism has implications for the prognosis of R‐ED, and a range of studies have documented that comorbid autism is associated with poorer outcomes of eating disorders across a range of treatment modalities (Nielsen et al., 2015, 2022; Tchanturia et al., 2019, Table 1).

**TABLE 1:** Comparing young persons (YPs) with Restrictive type eating disorders (R‐ED) and with or without autism

	YPs with autism	YPs without autism	Difference
N (%)	16 (10.2)	141 (89.8)	
Intake IEBW^a^ (mean, range, SD)	54.2, 32.5–68, 9.1	51.5, 29.5–79, 8.8	ns
Female sex assigned at birth (N, %)	13 (81.3)	131 (92.9)	ns
Age at beginning of treatment (mean, range, SD)	14.3, 11.5–17.3, 1.6	14.4, 10.3–17.5, 1.6	ns
Intensified care, N (%)	8 (50)	23 (16)	Difference in proportions: 0.34, *p* = 0.004
Weight normalisation^b^, N (%)	14 (87)	103 (73)	ns
Successful treatment	8 (50)	79 (56)	ns
Time to remission for those who ended the treatment successfully, median	9.1 months	9.2 months	ns

Abbreviations: IEBW, individual, expected body weight for each child or adolescent; ns, nonsignificant; YPs, young persons.

^a^ IEBW = mean expected body weight z‐score.

^b^ weight within 95% of IEBW by 12 months of treatment or EOT, whichever came first.

When treating YPs for R‐ED, family‐based treatment (FBT) is recommended as first choice of treatment (Wilson & Shafran, 2005). Family‐based treatment focuses on parent empowerment, supporting parents to kerb symptom behaviours as an expression of care for their ill child (Lock, 2018; Lock & Le Grange, 2015). Knowledge is sparse, however, on whether worse outcomes of R‐ED for YP with comorbid autism is true also in FBT. To the best of our knowledge, FBT in the context of autism has not been quantitatively evaluated. Interestingly, Loomes and Bryant‐Waugh recently proposed ways of accommodating FBT to YPs with autism (Loomes & Bryant‐Waugh, 2021). They recommend improving predictability of assessment and therapy sessions, consideration for the YP's sensory preferences, and YP's preference for routine and sameness in the renourishment process. In parallel, adaptions of evidence‐based treatments for other disorders in YPs with autism are emerging, for example, for co‐occurring anxiety or obsessive compulsive disorder, and show preliminary evidence of efficacy (Postorino et al., 2017; Vasa et al., 2014). Adaptations include increasing parent involvement to enhance generalisation, ‐ not unlike the guiding principle of FBT for R‐ED in YPs.

It would be clinically useful to understand whether the chance of weight normalisation and remission in FBT is similar for YPs with and without autism. Moreover, clinical experience suggests that YPs with autism and R‐ED require a longer period of time to achieve weight gain, to reach remission or both, and they more often need intensified treatment modalities such as day programme or inpatient treatment. In an earlier publication, we have evaluated outcomes within 12 months of FBT in a prospective, uncontrolled study of a consecutive series of patients with R‐ED, treated with FBT as standard care in a specialised outpatient unit at a government funded Child and Adolescent Mental Health Centre (Bentz et al., 2021). In the present short report, we wish to focus on outcomes of the subgroup of YPs with autism in thes earlier described series to better understand this subgroup's response to FBT. Moreover, we wish to discern what we can learn about their needs in R‐ED treatment and to inform hypotheses regarding potential improvements.

Consequently, we assessed whether YPs with autism more often were hospitalised or offered day programme treatment than YPs without autism. In addition, we evaluated weight normalisation, successful ending of treatment and the mean duration of successful treatment between groups with and without comorbid autism to evaluate the hypothesis that YPs with autism may need more time to benefit well from treatment.

The aim of this short report is to compare the groups with and without autism in a large consecutive series of YPs with R‐ED treated with FBT as the first type of intervention, regarding

- the proportion of YPs receiving intensified care,
- the proportion of YPs who obtained weight normalisation, and who successfully ended treatment at 12 months or at end of treatment (EOT), whichever came first,
- time to remission, that is, duration of treatment for those who successfully ended treatment.

## 2 METHODS

### 2.1 Participants

The unit receives all YPs under the age of 18 years who are referred for psychiatric treatment for an eating disorder in the geographical area of the capital region of Denmark. We included all patients with an ICD‐10 diagnosis of typical or atypical AN (F50.0 or F50.1) (WHO, 1992) during a period of 16 months if parents and patient gave informed consent. *A diagnosis of atypical AN implies a condition similar to typical AN*, *where one of the diagnostic criteria is not met*.

An autism diagnosis was registered if assigned either prior to or after diagnosis of R‐ED. Autism diagnoses were based on an Autism Diagnostic Observation Schedule with the YP and a thorough developmental history such as an Autism Diagnostic Interview with parents.

When diagnosed after the diagnosis of ED, assessment was initiated by the clinical need to understand the YP better, and not through a systematic screening of all participants.

### 2.2 Outcomes

Outcomes were weight normalisation and overall successful treatment. Weight normalisation was based on individual, expected body weight for each child or adolescent (IEBW) according to his/her childhood growth trajectory z‐score on weight‐for‐length/height, based on Danish norms (Tinggaard et al., 2014). Overall successful treatment represented the collaborative clinical decision made by the family and the therapist in the conversation towards the end of phase two, and in phase three. It reflects the appraisal that the YP is well, and that the family can manage potential residual symptoms without further treatment. We found that this aligns well with, but is not completely identical with more objective definitions of remission in a prior study of this cohort (Bentz et al., 2021). 77% of YPs who reached successful ending of the treatment in the total sample were weight restored defined as ≥95% of IEBW, 79% of those with successful ending of the treatment had fully resumed age‐appropriate responsibility for eating, 95% of them no longer reported intention of dietary restraint, 84% reported normalised levels of weight and shape concerns, and 75% of girls after menarche in this group had resumed menstruation.

### 2.3 Treatment

Treatment followed the manual by Lock and le Grange, albeit with few adaptations to fit local service context, as earlier described (Bentz et al., 2021). In accordance with the manual, families are not offered meal plans, but parents are guided regarding energy dense foods and are advised to rely on their knowledge of their child and their family culture when planning meals. Inpatient stay or a day programme was available and was offered in case of somatic or psychiatric risk or in case of a lack of progress for several months; intensified treatment might also be suggested by the therapist or team and/or the family. These treatment modalities include supported family meal training, and thus involve parent empowerment in line with the principles of FBT. In the prior study we reported that 20% of the total sample spent time in the inpatient or day programme services.

### 2.4 Statistics

Data was managed using REDCap electronic data capture tools hosted at The Capital Region of Denmark (Harris et al., 2009, 2019), and data was processed using Statistical Package for the Social Sciences, ver. 25 (IBM, 2019). Due to the small sample size of the autism group, Fisher's exact test was run to test the difference of proportions in intensified care and proportions with weight normalisation by 12 months. Difference in proportions of successful ending of the treatment by 12 months was analysed with chi‐square test of homogeneity. The difference in duration of successful ED treatment was analysed with Independent‐Samples Mann‐Whitney *U* test due to the presence of outliers in both groups.

## 3 RESULTS

A total of 167 YPs (73% of eligible patients in this period) gave consent. Ten moved out of the region or were referred to adult mental health services before 12 months, leaving a sample of 157 YPs. Diagnoses were typical AN (F50.0) in 97 (61.8%) of participants, and atypical AN in 60 (38.2%). 10.2% (*N* = 16) of the sample had autism. 2.5% (*N* = 4) had autism diagnosed prior to the ED, and an additional 7.7% (*N* = 12) were diagnosed with autism during ED treatment. The autism and the non‐autism group had comparable degrees of underweight at the beginning of treatment.

### 3.1 Aim a: intensified care

A significantly larger proportion of YPs with autism (50%) received intensified care at some point during their treatment, compared with YPs without autism (16%).

### 3.2 Aim b: weight normalisation and successful ending of treatment by 12 months

We found no statistically significant difference in proportions between groups regarding weight normalisation nor successful ending of treatment.

In parallel, there was no statistically significant difference between groups regarding successfully ended treatment by 12 months of treatment or EOT, whichever came first.

### 3.3 Aim c: time to remission

Of the total sample, 67% (*N* = 109) ended treatment successfully. Median treatment duration for those who reached successful ending of the treatment was around 9 months and was not statistically different between participants with autism and participants without autism.

## 4 DISCUSSION

The total proportion of YPs with autism in this sample is within the range of 4%–10% documented in prior studies on YPs with AN (Westwood & Tchanturia, 2017). As expected, it is higher than the proportion seen in the general population.

Comparable proportions of YPs with and without autism restored normal weight and ended treatment successfully within 12 months, and they did so at the same speed as those without autism, despite earlier findings of poorer outcomes of R‐ED in the context of autism. For instance, in a long‐term follow‐up study of YPs with R‐ED in adolescence, the proportion of recovery was significantly lower among the YPs who had co‐occurring autism (Nielsen et al., 2015, 2022). If confirmed in a larger sample, our finding of comparable outcomes between groups might in part be associated with the strong involvement of parents in FBT, a treatment that was not implemented at the time of the longitudinal study.

However, for significantly more YPs with autism, outpatient management was not deemed sufficient, as they had a period of more intensive intervention (day programme or inpatient treatment). Thus, for half of the YPs with autism, outpatient FBT was not able to bring about necessary change. Outpatient FBT thus may have lacked something required in these cases, and to understand what that requirement is, might point to improvements that could keep more YPs with autism in outpatient care in the future, rather than needing intensified treatment. For instance, a highly structured environment and meal plans might be important differences between outpatient FBT and intensified treatment, that were beneficial for the YP's with autism who recieved intensified treatment. Unfortunately, we do not have data to suggest what the primary purpose of intensification was, for example, whether intensification of treatment was chosen with the primary purpose to establish regular and sufficient eating, or to modify interactions around meals, including for example practicing skills to manage intense emotional reactions. Future studies may focus on understanding these processes better. It is possible that following suggestions from Loomes and Bryant‐Waugh regarding adapting FBT to those with autism (Loomes & Bryant‐Waugh, 2021), or the suggestions of the Pathway for eating disorders and autism pathway for autism friendly service organisation (Tchanturia et al., 2020) might increase the proportion of successful outpatient FBT for YPs with autism. Moreover, as parents are the primary agents of change in the first phases of FBT, meeting the needs of parents may be especially important when treating YPs with R‐ED and autism. Two qualitative studies found tearers in this situation experienced distress and lack of support in eating disorder services (Adamson et al., 2020; Kinnaird et al., 2021). It is feasible that specialised support for carers for a loved one with autism and ED may benefit not only the carers but the YP with R‐ED and autism as well. This need is strengthened by the fact that majority of YPs with autism in our sample recieved their autism diagnosis while in R‐ED treatment, and consequently these parents and YPs had to come to terms with the implications of this new diagnosis while simultaneously managing the struggle for renourishment.

On the other hand, another half of the YPs with autism seemed to benefit well from standard outpatient FBT. We speculate that especially the well‐structured renourishment phase (phase one) with parental leave and clear treatment targets may suit these YPs well. Predictability and consideration for sensory or routine preferences are not an explicit part of the FBT manual, but parents in FBT are supported to implement renourishment and support in a way that is congruent with their family style, and these parents may be experienced in attuning to their particular child's individual need for structure and predictability (while treatment staff are not). Some parents have prior experience with structuring their child's eating pattern in the face of difficulty; for instance, a recent epidemiological study demonstrated, that a subgroup of YPs with autism spectrum traits had fussy, selective eating prior to developing a R‐ED (Carter Leno et al., 2022). Possibly, there might be a dual pathway where one proportion of YPs with autism quickly change their focus, for example, from restriction to regularity in response to the well‐structured support from parents and treatment, whereas another proportion may enter a vicious cycle of frustration, power struggles or emotional dysregulation, rendering parents and outpatient FBT powerless. Unfortunately, our sample size does not allow stratification and separate analyses of potential characteristics to separate these groups.

The findings of this short report need confirmation in a larger sample. If confirmed, potential implications for adapting FBT to the subgroup of YPs with autism may be a format of FBT that from the start involves a very literal, concrete, and predicable style, with written materials, suggested meal plans, and well‐defined negotiables and non‐negotiables in order to reduce stress and increase the chance of success.

## 5 LIMITATIONS

The present study is exploratory, with a small autism sample, and with no correction for multiple comparisons, and consequently, findings are preliminary. Moreover, most autism diagnoses in the sample were assigned after R‐ED, and this implies two related limitations; first typically assessment for autism is only initiated when treatment is difficult, which may explain why more YPs with autism receive intensified treatment. Second, autism traits are distributed within the AN population (Rhind et al., 2014), and a screening for autism for all participants may have better informed how these traits impact response to FBT.

## 6 CONCLUSION

Preliminary findings suggest that YPs with autism offered FBT as the first choice of treatment for R‐ED seem to normalise weight and end treatment successfully at comparable rates as YPs without autism. However, half of those with autism needed more intensive forms of treatment than outpatient FBT. A larger sample size of YPs with autism is needed to analyse differences between those who succeed with outpatient FBT alone and those who need intensification. Data from this short report indicate that FBT may be a reasonable first line of treatment for a subgroup of YPs with autism and R‐ED, but more knowledge and replication in a larger sample is needed, to understand different pathways for different subgroups of YPs with autism. Further, rates of success in the future may be improved by developing autism‐friendly adaptations to FBT.

**Abbreviations**
ADOS‐2: Autism Diagnostic Observation Schedule
ADI‐R: Autism Diagnostic Interview
AN: Anorexia Nervosa
EOT: end of treatment
ED: eating disorder
FBT: Family‐based treatment
ICD: International Classification of Diseases
IEBW: individual, expected body weight for each child or adolescent
N: number; OCD, Obsessive Compulsive Disorder; PEACE Pathway, Pathway for Eating Disorders and Autism
R‐ED: Restrictive type eating disorders
YP: young person

## CONFLICT OF INTEREST

Authors declare no conflicts of interest.

## Data Availability

The data that support the findings of this study are available from the corresponding author upon reasonable request.

## References

[erv2938-bib-0001] Adamson, J. , Kinnaird, E. , Glennon, D. , Oakley, M. , & Tchanturia, K. (2020). Carers’ views on autism and eating disorders comorbidity: Qualitative study. BJPsych Open, 6(3). 10.1192/bjo.2020.36 PMC7331083 32419683

[erv2938-bib-0002] Autism Europe . (2022). Acceptable language. Retrieved from https://www.autismeurope.org/about‐autism/acceptable‐language/

[erv2938-bib-0003] Bentz, M. , Pedersen, S. H. , & Moslet, U. (2021). An evaluation of family‐based treatment for restrictive‐type eating disorders, delivered as standard care in a public mental health service. Journal of Eating Disorders, 9(1), 141. 10.1186/s40337-021-00498-2 34715920 PMC8555240

[erv2938-bib-0004] Brede, J. , Babb, C. , Jones, C. , Elliott, M. , Zanker, C. , Tchanturia, K. , Serpell, L. , Fox, J. , & Mandy, W. (2020). For me, the anorexia is just a symptom, and the cause is the autism”: Investigating restrictive eating disorders in autistic women. Journal of Autism and Developmental Disorders, 50, 1–17. 10.1007/s10803-020-04479-3 32274604 PMC7677288

[erv2938-bib-0005] Carter Leno, V. , Micali, N. , Bryant‐Waugh, R. , & Herle, M. (2022). Associations between childhood autistic traits and adolescent eating disorder behaviours are partially mediated by fussy eating. European Eating Disorders Review. 10.1002/erv.2902 PMC9542277 35388530

[erv2938-bib-0006] Harris, P. A. , Taylor, R. , Minor, B. L. , Elliott, V. , Fernandez, M. , O’Neal, L. , McLeod, L. , Delacqua, G. , Delacqua, F. , Kirby, J. , & Duda, S. N. (2019). The RED Cap consortium: Building an international community of software platform partners. Journal of Biomedical Informatics, 95, 103208. 10.1016/j.jbi.2019.103208 31078660 PMC7254481

[erv2938-bib-0007] Harris, P. A. , Taylor, R. , Thielke, R. , Payne, J. , Gonzalez, N. , & Conde, J. G. (2009). Research electronic data capture (REDCap)—A metadata‐driven methodology and workflow process for providing translational research informatics support. Journal of Biomedical Informatics, 42(2), 377–381. 10.1016/j.jbi.2008.08.010 18929686 PMC2700030

[erv2938-bib-0008] Huke, V. , Turk, J. , Saeidi, S. , Kent, A. , & Morgan, J. F. (2013). Autism spectrum disorders in eating disorder populations: A systematic review. European Eating Disorders Review, 21(5), 345–351. 10.1002/erv.2244 23900859

[erv2938-bib-0009] IBM, Corp . (2019). IBM SPSS statistics for macintosh, version 25.0. IBM Corp.

[erv2938-bib-0010] Kinnaird, E. , Oakley, M. , Lawrence, V. , Shergill, S. , & Tchanturia, K. (2021). A peer interview qualitative study exploring support for carers of people with comorbid autism and eating disorders. Journal of Eating Disorders, 9(1), 42. 10.1186/s40337-021-00397-6 33789761 PMC8010292

[erv2938-bib-0011] Lock, J. (2018). Family therapy for eating disorders in youth: Current confusions, advances, and new directions. Current Opinion in Psychiatry, 31(6), 431–435. 10.1097/YCO.0000000000000451 30063479

[erv2938-bib-0012] Lock, J. , & Le Grange, D. (2015). Treatment manual for anorexia nervosa. In A family‐based approach (2nd ed.). Guilford Publications.

[erv2938-bib-0013] Loomes, R. , & Bryant‐Waugh, R. (2021). Widening the reach of family‐based interventions for anorexia nervosa: Autism‐adaptations for children and adolescents. Journal of Eating Disorders, 9(1), 1–11. 10.1186/s40337-021-00511-8 34863292 PMC8645124

[erv2938-bib-0014] Nielsen, S. , Anckarsäter, H. , Gillberg, I. C. , Gillberg, C. , Råstam, M. , & Wentz, E. (2015). Effects of autism spectrum disorders on outcome in teenage‐onset anorexia nervosa evaluated by the Morgan‐Russell outcome assessment schedule: A controlled community‐based study. Molecular Autism, 6(1), 14. 10.1186/s13229-015-0013-4 25774282 PMC4359566

[erv2938-bib-0015] Nielsen, S. , Dobrescu, S. R. , Dinkler, L. , Gillberg, C. , Gillberg, C. , Råstam, M. , & Wentz, E. (2022). Effects of autism on 30‐year outcome of anorexia nervosa. Journal of Eating Disorders, 10(1), 4. 10.1186/s40337-021-00518-1 35000620 PMC8744255

[erv2938-bib-0016] Postorino, V. , Kerns, C. M. , Vivanti, G. , Bradshaw, J. , Siracusano, M. , & Mazzone, L. (2017). Anxiety disorders and obsessive‐compulsive disorder in individuals with autism spectrum disorder. Current Psychiatry Reports, 19(12), 92. 10.1007/s11920-017-0846-y 29082426 PMC5846200

[erv2938-bib-0017] Rhind, C. , Bonfioli, E. , Hibbs, R. , Goddard, E. , Macdonald, P. , Gowers, S. , Schmidt, U. , Tchanturia, K. , Micali, N. , & Treasure, J. (2014). An examination of autism spectrum traits in adolescents with anorexia nervosa and their parents. Molecular Autism, 5(1), 56. 10.1186/2040-2392-5-56 25553237 PMC4280745

[erv2938-bib-0018] Steinhausen, H.‐C. , & Jensen, C. M. (2015). Time trends in lifetime incidence rates of first‐time diagnosed anorexia nervosa and bulimia nervosa across 16 years in a Danish nationwide psychiatric registry study. International Journal of Eating Disorders, 48(7), 845–850. 10.1002/eat.22402 25809026

[erv2938-bib-0019] Tchanturia, K. , Adamson, J. , Leppanen, J. , & Westwood, H. (2019). Characteristics of autism spectrum disorder in anorexia nervosa: A naturalistic study in an inpatient treatment programme. Autism, 23(1), 123–130. 10.1177/1362361317722431 29105513

[erv2938-bib-0020] Tchanturia, K. , Smith, K. , Glennon, D. , & Burhouse, A. (2020). Towards an improved understanding of the anorexia nervosa and autism spectrum comorbidity: PEACE pathway implementation. Frontiers in Psychiatry, 11. 10.3389/fpsyt.2020.00640 PMC7358367 32733294

[erv2938-bib-0021] Tinggaard, J. , Aksglaede, L. , Sørensen, K. , Mouritsen, A. , Wohlfahrt‐Veje, C. , Hagen, C. P. , Mieritz, M. G. , Jørgensen, N. , Wolthers, O. D. , Heuck, C. , Petersen, J. H. , Main, K. M. , & Juul, A. (2014). The 2014 Danish references from birth to 20 years for height, weight and body mass index. Acta Paediatrica, 103(2), 214–224. 10.1111/apa.12468 24127859

[erv2938-bib-0022] Vasa, R. A. , Carroll, L. M. , Nozzolillo, A. A. , Mahajan, R. , Mazurek, M. O. , Bennett, A. E. , Wink, L. K. , & Bernal, M. P. (2014). A systematic review of treatments for anxiety in youth with autism spectrum disorders. Journal of Autism and Developmental Disorders, 44(12), 3215–3229. 10.1007/s10803-014-2184-9 25070468

[erv2938-bib-0023] Westwood, H. , & Tchanturia, K. (2017). Autism spectrum disorder in anorexia nervosa: An updated literature review. Current Psychiatry Reports, 19(7). 10.1007/s11920-017-0791-9 PMC5443871 28540593

[erv2938-bib-0024] WHO . (1992). The ICD‐10 classification of mental and behavioural disorders: Clinical descriptions and diagnostic guidelines. World Health Organization.

[erv2938-bib-0025] WHO . (2021). ICD‐11—ICD‐11 for mortality and morbidity statistics. Retrieved from https://icd.who.int/browse11/l‐m/en

[erv2938-bib-0026] Wilson, G. T. , & Shafran, R. (2005). Eating disorders guidelines from NICE. The Lancet, 365(9453), 79–81. 10.1016/S0140-6736(04)17669-1 15639682

